# The HIV-1 Envelope Transmembrane Domain Binds TLR2 through a Distinct Dimerization Motif and Inhibits TLR2-Mediated Responses

**DOI:** 10.1371/journal.ppat.1004248

**Published:** 2014-08-14

**Authors:** Eliran Moshe Reuven, Mohammad Ali, Etai Rotem, Roland Schwarzter, Andrea Gramatica, Anthony H. Futerman, Yechiel Shai

**Affiliations:** 1 Department of Biological Chemistry, The Weizmann Institute of Science, Rehovot, Israel; 2 Institut für Biologie, AG Molekulare Biophysik, Humboldt-Universität zu Berlin, Berlin, Germany; Vaccine Research Center, United States of America

## Abstract

HIV-1 uses a number of means to manipulate the immune system, to avoid recognition and to highjack signaling pathways. HIV-1 infected cells show limited Toll-Like Receptor (TLR) responsiveness via as yet unknown mechanisms. Using biochemical and biophysical approaches, we demonstrate that the trans-membrane domain (TMD) of the HIV-1 envelope (ENV) directly interacts with TLR2 TMD within the membrane milieu. This interaction attenuates TNFα, IL-6 and MCP-1 secretion in macrophages, induced by natural ligands of TLR2 both in *in vitro* and *in vivo* models. This was associated with decreased levels of ERK phosphorylation. Furthermore, mutagenesis demonstrated the importance of a conserved GxxxG motif in driving this interaction within the membrane milieu. The administration of the ENV TMD *in vivo* to lipotechoic acid (LTA)/Galactosamine-mediated septic mice resulted in a significant decrease in mortality and in tissue damage, due to the weakening of systemic macrophage activation. Our findings suggest that the TMD of ENV is involved in modulation of the innate immune response during HIV infection. Furthermore, due to the high functional homology of viral ENV proteins this function may be a general character of viral-induced immune modulation.

## Author Summary

To understand viral pathology and the tools needed to eliminate infection, it is important to understand how viral immune evasion occurs. One such mode of inhibition is the decreased responsiveness of Toll-Like Receptors (TLRs). To date, the exact mechanism inducing this inhibition is not clear. In this study, we utilized a multidisciplinary approach and report on direct modulation of TLR2 activity by the envelope trans-membrane protein of HIV-1 through trans-membrane domain interactions. This interaction resulted in a decreased response *in vitro* of TLR2 to its natural ligand LTA. Through mutagenesis analysis we show that the GxxxG motif is the driving force of this interaction. Interestingly, the inhibitory effect was also highly effective in protecting mice from lethal effects in a sepsis-like model. Our findings implicate that ENV participates in innate immune impairment, which may occur during viral entry and at latent stages. Furthermore, due to the high functional homology between viral ENV proteins, this function may exhibit a general character of viral-induced immune modulation.

## Introduction

The ongoing race between pathogens and their hosts' responses to eliminate them led to the development of many mechanisms driven by the invading pathogen to impair immune responses. The cell populations that are mainly targeted by the virus include mononuclear phagocytes (e.g. macrophages and monocytes) and T cells [1]. Several mechanisms of immune evasion and suppression have been described for the pathology of the human immunodeficiency virus type 1 (HIV-1) [2], [3]. Regarding mononuclear phagocytes, studies implicated the importance of early genes, expressed by HIV-1 in advanced phases of infection, for immune manipulation [4], [5]. However, as these cells are hallmarks of innate immunity, there is also a requirement for immune manipulation at stages of viral entry and latency. Little is known about the ability of HIV-1 to modulate innate immune responses of these cells during its entry and latent stages, particularly against members of the Toll-Like receptor (TLR) family.

TLRs are critical in the immediate innate immune response against bacterial and viral pathogens [6], [7]. TLRs are conserved membrane receptors that recognize a wide variety of pathogen-associated molecular patterns (PAMPs), such as lipopolysaccharide (LPS) from Gram-negative bacteria, lipoteichoic acid (LTA) from Gram-positive bacteria, flagellin, in addition to intracellular molecules such as single-stranded DNA and RNA [8], [9]. To induce ligand recognition and subsequent signaling, the hetero-dimerization of TLR2 with TLR6 or TLR1 is required. This is coordinated through ligand binding to the extracellular regions of the proteins and conformational changes throughout the proteins [10], [11], [12]. The significance of the TLR2 and TLR6 TMDs in the regulation and activation of formation of the receptor complex and in downstream signaling has been recently described [13], revealing that activation of TLR2 increases resistance of macrophages to HIV-1 infection [14]. Interestingly, dendritic cells (DCs) infected with HIV-1 were reported to be less responsive via TLR upon expression of ENV on the membrane [15]. These emerging studies link the manipulation of TLR2 responses and HIV-1 pathogenesis through as yet unknown mechanisms.

HIV-1 infects cells via the HIV-1 ENV protein which mediates viral entry to host cells that express CD4 together with an additional co-receptor such as monocytes and dendritic cells, through membrane fusion. In addition to its fusogenic activity, the ENV protein binds proteins localized to membrane microdomains on macrophages and DCs. In addition, TLRs expressed on the cell membrane are recruited to cholesterol-enriched membrane microdomains upon their ligand recognition, initiating signal transduction [16], [17]. ENV is also targeted to cholesterol-enriched membrane microdomains through a well-defined localization signal located adjacent to its TMD. The TMD of ENV and its adjacent regions are extremely conserved among all clades currently reported of HIV-1, and are similar to the TMD of HIV-2 gp41 [18]. Interestingly, recent studies showed that TLR2 recognition of several viral related glycoproteins (gp) induces activation of an antiviral response [19]. Taken together, we hypothesized that the ENV plays a role in impairment of TLR-induced responses, contributing both to its evasion from recognition during the fusion process and to expropriate native TLR responses at latent stages, thus assisting in viral replication upon its initiation.

Here, we report that the gp41 TMD associates with TLR2 TMD in the membrane. As a result, cultured macrophages treated with peptides derived from the gp41 TMD displayed reduced TLR2-mediated signaling and decreased pro-inflammatory cytokine secretion. Moreover, the ectopic expression of the intact gp41 and gp160 inhibited LTA mediated cytokine secretion with gp41 having the highest potency. To demonstrate the TLR2 inhibitory effect i*n vivo*, we show that the gp41 TMD protected mice against LTA and D-galactosamine (LTA/GLN)-mediated acute sepsis, concomitant with a decrease in tissue damage.

## Results

### Gp41 TMD Inhibits TLR2/6 Signaling and Pro-inflammatory Gene Expression in RAW264.7 Cells

**Figure 1:**
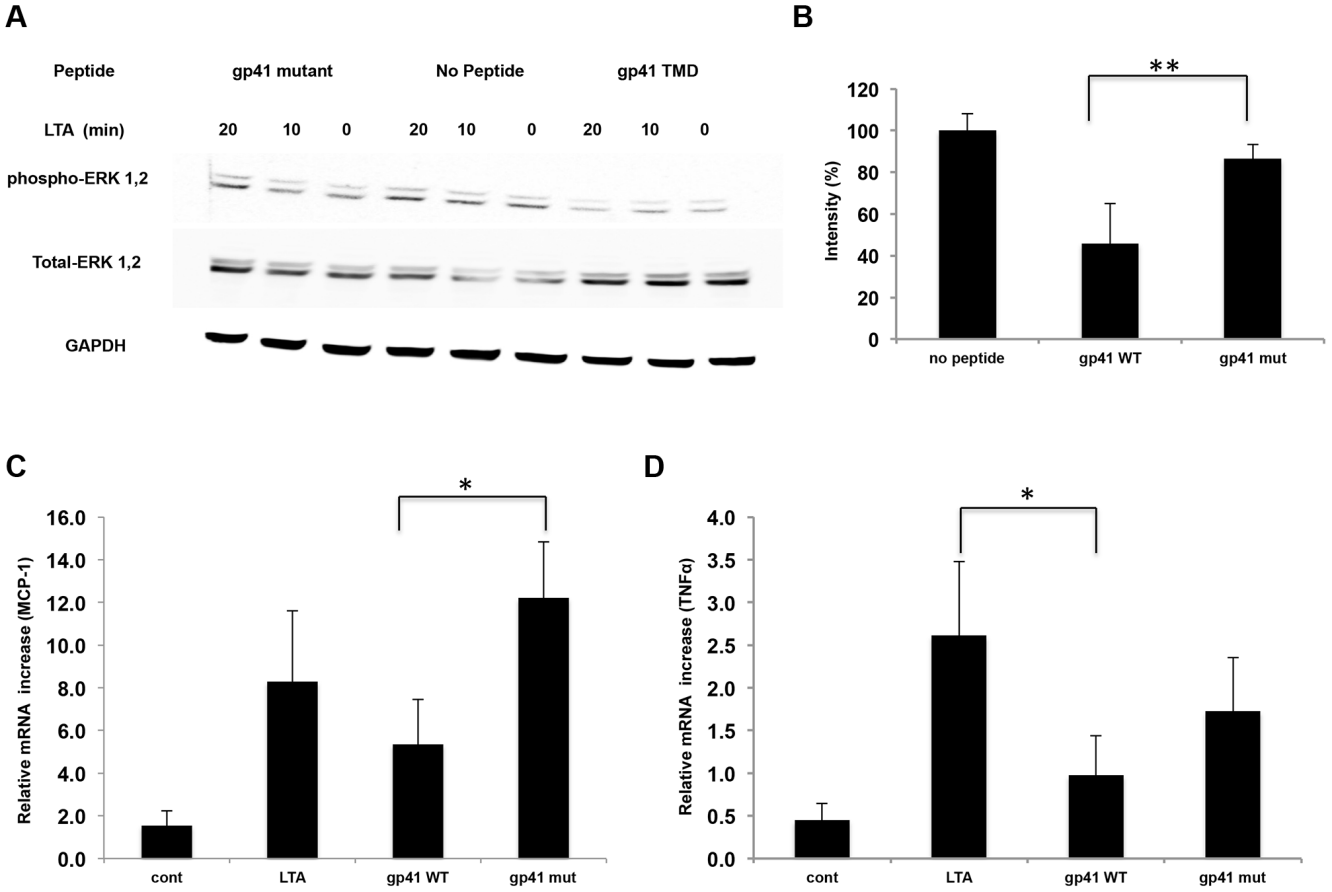
The gp41 TMD inhibits TLR2 induced signaling through ERK1,2 and cytokine secretion. (**A**) ERK1,2 phosphorylation in RAW264.7 cells was detected before and after LTA exposure. Cells were either pre-treated with indicated peptides for 2 hours prior to LTA addition or untreated (center). GAPDH shows equal loading. Here shown representative data of 3 typical experiments. (**B**) Quantification of the band intensity was determined for the p-ERK levels after 20 minutes LTA exposure. The WT peptide shows over 50 minutes LTA exposure. The WT peptide shows over 50% decrease in p-ERK levels while the mutant peptide shows approximately 15%. Statistical analysis was done by student T-test, (n = 4, ** p<0.01). (**C–D**) mRNA expression levels of MCP-1 (C) and TNFα (D) detected by real-time PCR. Expression levels were normalized to the expression of non-activated cells. The data is shown as the calculated 2^∧−dCT^ in the relativity of each gene to HPRT. (The figure is an average of three samples, * - p<0.05).

We synthesized a peptide derived from the N′ terminus of the ENV TMD region (gp41 TMD) previously reported to be involved in cellular responses to viral infection [20]. Since this region harbors the well-defined GxxxG motif known to drive the assembly of TMDs [21], we also synthesized a peptide lacking this motif (gp41 mutant) (Table S1 in Text S1). We then utilized the macrophage cell line RAW264.7 to study the functionality of TLR2. We activated TLR2 by purified LTA that was recently shown to be regulated, at least in part, through interactions between the TMDs of TLR2 and TLR6 [13]. We measured the level of phosphorylated ERK1,2 upon treatment with LTA, as these are key regulators of the TLR signaling pathway. Figure 1A and B show that 20 minutes after addition of LTA to RAW cells that were pre-incubated with the gp41 TMD peptide, there was a significant decrease in ERK1,2 phosphorylation compared to non-treated cells, indicating less receptor activation. As a negative control, pre-incubation with the gp41 mutant peptide did not change the levels of phosphorylated ERK1,2. Noteworthy, since the cells in culture that respond through the TLRs also respond to endogenous signals, the basal level of phosphorylated ERK1,2 was also decreased by the gp41 TMD peptide but not by the mutant peptide (Figure 1A). We next measured the expression levels of NFκB downstream genes; Tumor Necrosis Factor α (TNFα), a hallmark cytokine of TLR activation, and Monocyte Chemotactic Protein 1 (MCP-1), a major chemokine mainly secreted by macrophages [22], in order to ensure that the inhibitive effect on ERK1,2 phosphorylation is a result of inhibition of TLR2 signaling. Treatment of the cells with gp41 TMD peptide resulted in a 45% decrease in MCP1 mRNA expression levels compared to non-treated cells (Figure 1C). The treatment also resulted in a 72% decrease in the mRNA expression levels of TNFα (Figure 1D). When cells were treated with the mutant peptide, only a 34% decrease in mRNA levels was detected for TNFα (Figure 1C), and no significant change was detected for the expression levels of MCP-1 (Figure 1D). Altogether, it is clear that the association between the TMDs of gp41 and TLR2 functionally impacts receptor activity.

**Figure 2:**
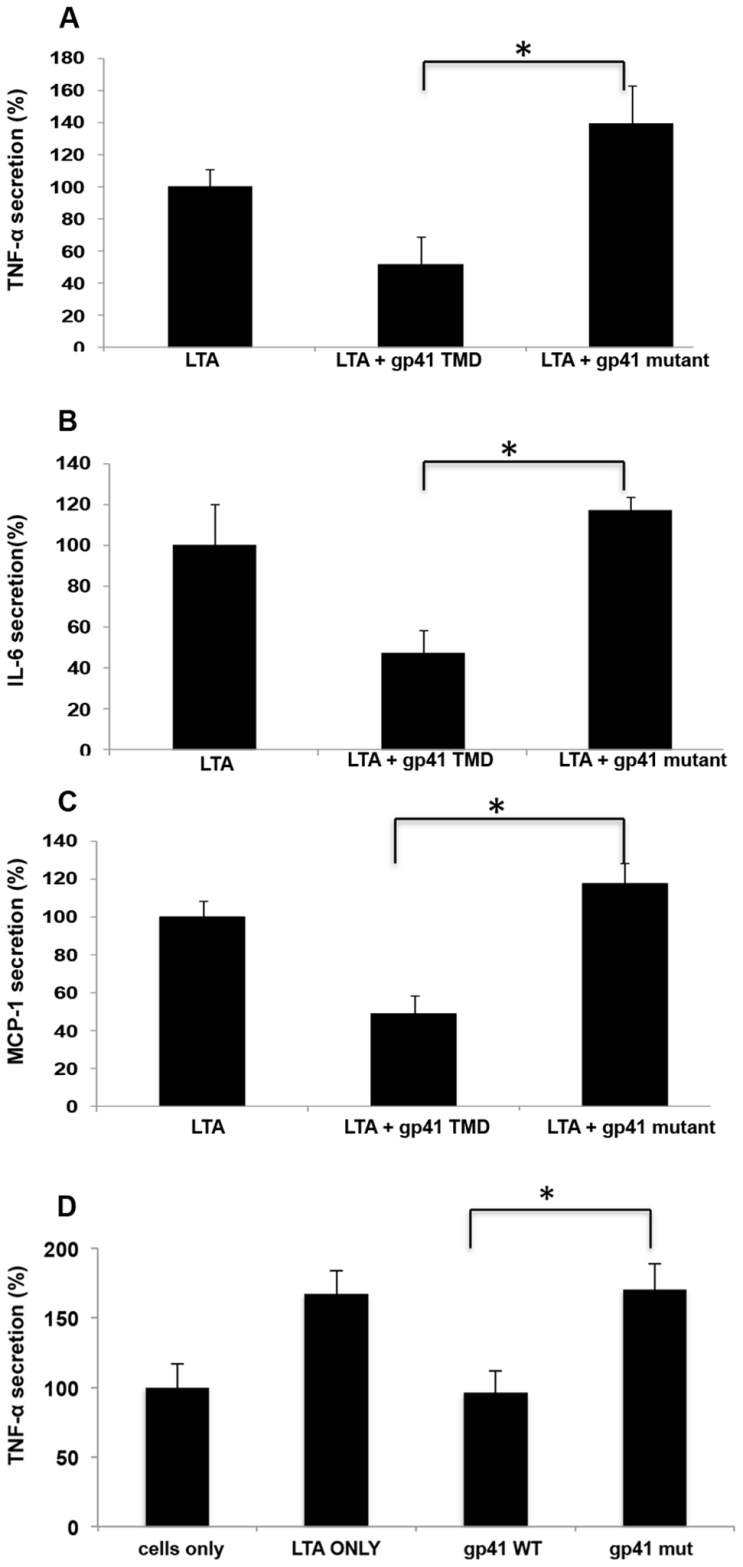
ENV TMD inhibits secretion of cytokines and chemokines induced by TLR2. (**A–C**) RAW cells were incubated with/without the indicated peptides for 2 hours, then washed twice and activated with 500 ng hours, then washed twice and activated with 500 ng ng/ml of purified LTA. Media was collected after 5 hours (TNFα) or 24 hours (MCP-1 and IL-6). ELISA was performed using standard kits. (n = 3, ± represent SD) (**D**) THP-1 human macrophages were incubated using similar conditions as described in A, and TNFα levels were measured (* - p<0.05).

In order to confirm that the effects observed at the transcription levels influence the protein expression levels we measured TNFα, MCP-1 and IL-6 secretion levels. IL-6 is an additional target gene of TLR2 but it is transcribed via a different transcription factor complex than TNFα. Cells were pre-incubated with ENV TMD peptides for two hours prior to LTA activation. Cytokine expression was measured in accordance to the expected time of expression of the various targets. The results show that the ENVTMD peptide attenuates the secretion of TNFα (Figure 2A), IL-6 (Figure 2B) and MCP-1 (Figure 2C). However, and in line with the mRNA expression levels and phosphorylation inhibition, the gp41 mutant peptide did not decrease cytokine secretion. In order to validate that the effect observed is not limited to murine cells we evaluated the effect of the peptides on human THP-1 cells. We followed the secretion of TNFα under the same conditions as for the mice cell line. The data show that the WT peptide had similar effect on human cells as in mice cells and that the mutant peptide did not affect the secretion levels of TNFα (Figure 2D). These results suggest that incubation of gp41 TMD peptides with cells leads to inhibition of TLR2/6 signaling. Together, these results suggest a potential potent immunosuppressive effect of gp41 HIV-1 ENV protein via inhibition of TLR2 mediated signaling.

**Figure 3:**
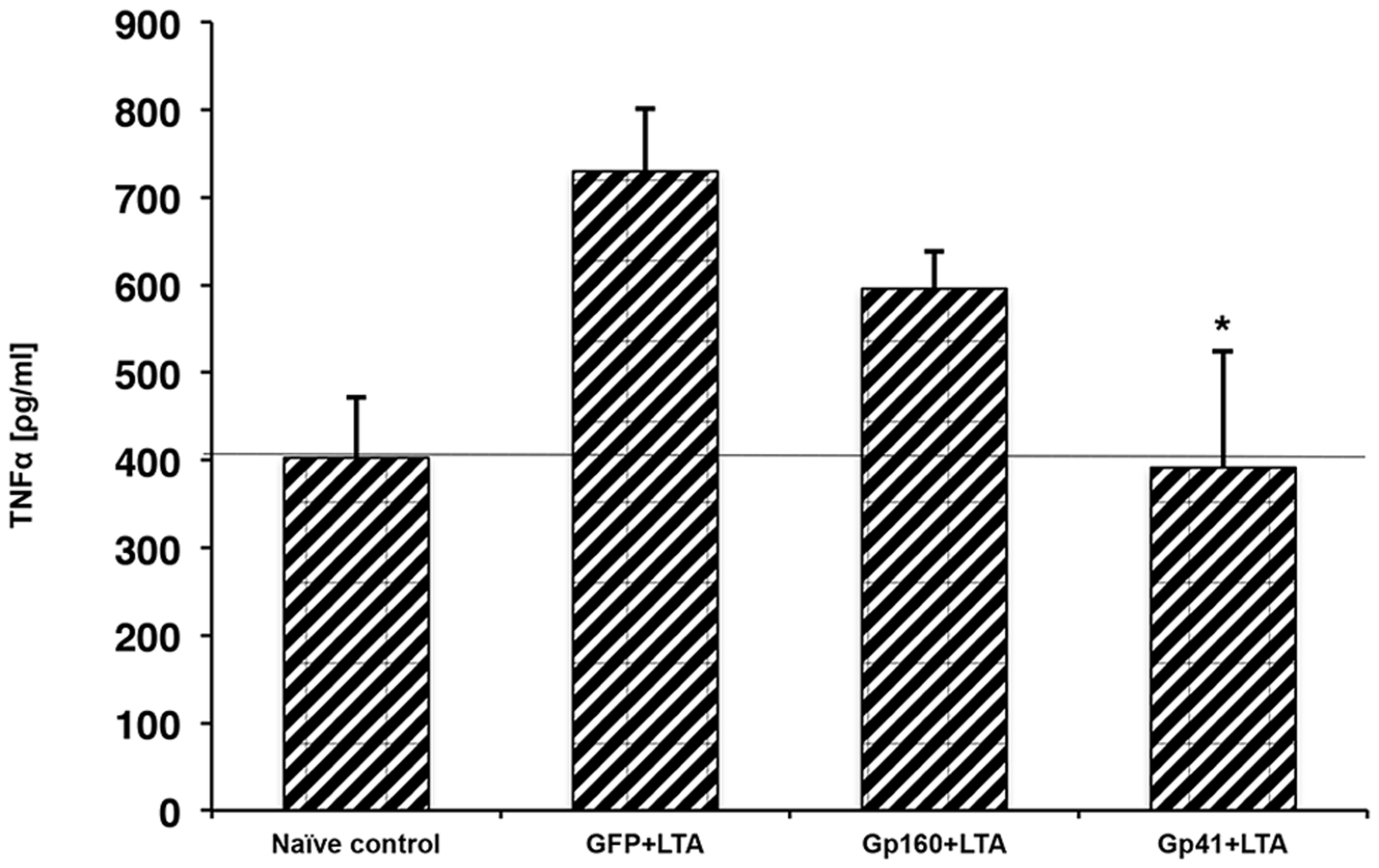
HIV1-ENV intact protein inhibits LTA mediated TNFα secretion. RAW cells were transfected either with vector plasmid (YFP) or gp160 HIV-1 ENV expressing plasmid or gp41 HIV-1 ENV expressing plasmid as indicated in materials and methods section, and after 48 hours of transfection cells were activated with 500 ng hours of transfection cells were activated with 500 ng ng/ml of purified LTA, and TNFα secretion levels were measured by ELISA and compared to non-treated RAW (naïve) cells. Results are from 3 typical experiments and each is a triplicate, *, p<0.05.

In order to confirm this hypothesis we utilized the ectopic expression of HIV-1 ENV-YFP chimeric protein in RAW 264.7 cells, which mimics the HIV-1 infection of macrophages, and tested their responsiveness to LTA by measuring TNFα secretion. We expressed two forms of the ENV protein, the full length gp160 and the transmembrane protein gp41. LTA responsiveness was compared to mock transfected cells (see materials and methods). LTA treatment resulted in elevated TNFα secretion in mock cells, whereas gp160 expressing cells showed a reduction in TNFα secretion. Furthermore, gp41 expressing cells showed even more reduction of TNFα secretion almost to the basal level of the non-treated cells (Figure 3). The better efficiency of gp41 in inhibiting TNFα secretion compared to gp160 can be in part due to the smaller size of its ectodomain compared to that of gp160. This should result in less hindrance upon its interaction with TLR2. We confirmed similar expression levels of HIV-1 gp160 and gp41, as they showed similar extracellular expression levels (see example in supplementary Figure S5 in Text S1). These data reveal that the fully processed ENV protein, once expressed on the cellular membrane, results in inhibition of TLR2 activation.

### The TMDs of Gp41 and TLR2 Interact within the Membrane, Involving the GxxxG Motif

**Figure 4:**
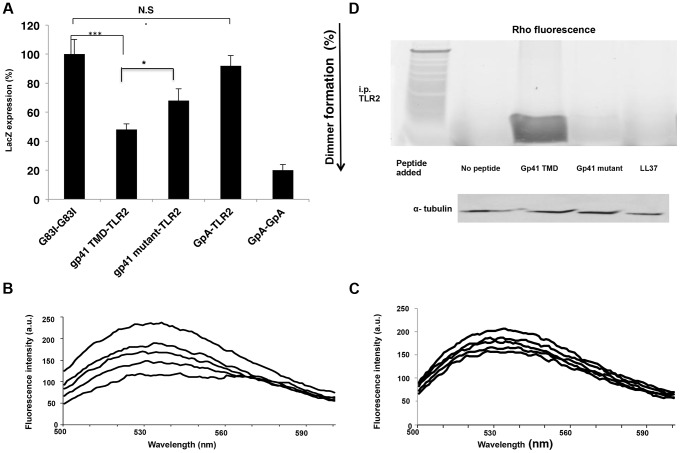
The gp41 TMD interacts directly with the TLR2 TMD *in vitro*, partially through its GxxxG dimmerization motif. (**A**) GALLEX assay reveals the interaction of the gp41 TMD with the TLR2 TMD. GpA and G83I were used as positive and negative controls of interaction, respectively. (n = 3, **-p<0.005, ***-p<0.0005). (**B–C**) FRET analysis of the interaction between the gp41 TMD (0.1 µM) (B) and gp41 mutant (0.1 µM) (C) peptides with the TLR2 TMD peptide in LUVs. Highest line represents fluorescence of the NBD peptide in the absence of acceptor peptides. Titration with successive amounts of acceptor peptides was performed from at 1∶40, 1∶20, 1∶10 and 1∶1 acceptor∶donor ratios. (D) co-immunoprecipitation (Co-i.p.) of Rho labeled peptides together with TLR2 proteins. 1*10^∧6^ cells were incubated for 2 hours with the indicated peptides and then cells were lysed and the Co hours with the indicated peptides and then cells were lysed and the Co-i.p. was performed. Band detection was performed by using a fluorescence spectra-photometer scanner. Figure is a representative of 3 independent experiments.

We studied the ability of the ENV TMD to target the TLR2 TMD as it naturally resides in the membrane, and the TMDs of TLR2/6 were shown to be important for their regulation and function. The GALLEX system [23], [24] was utilized to evaluate the hetero-association of gp41 and TLR2 TMDs in biological membranes. β-gal activity was measured after the expression of LexA-TMD-MBP chimera proteins in which the TMDs are those of ENV (wild type and mutant) and of TLR2. The GpA TMD sequence and its non-assembling mutant G83I (Table S1 in Text S1) served as positive and negative controls, respectively. Strong dimerization results in low β-gal activity. The level of association between the TMDs of gp41 WT and TLR2 was significantly higher than that of the TMDs of the gp41 mutant and TLR2. Moreover, there was no detectable association between the TMDs of TLR2 and GpA (containing the GxxxG motif) (Figure 4A). Therefore, we conclude that the GxxxG motif contributes but is not sufficient for the assembly of the TMDs of gp41 and TLR2.

FRET analysis was used to explore the direct interaction between the TMD peptides within phospholipid membranes (PC∶Chol 9∶1). The decrease in the emission of NBD conjugated peptides (gp41 TMD/mutant), after the addition of successive amounts of Rho-labeled TLR2 TMD, served as an indication of the interaction between a pair of peptides. The gp41 TMD peptide showed strong interaction with the TLR2 TMD peptide (Figure 4B). This is evident from the large decrease in the emission signal at 530 nm after the first addition of the Rho-TLR2 TMD peptide and by the dose dependent extent of association resulting in a decrease of over 60% at a 1∶1 ratio. In comparison, the interaction between the gp41 mutant and TLR2 TMDs peptides was significantly lower (less than 25%) (Figure 4C).

To determine the degree of interaction between the TMDs of ENV and TLR2 within macrophages we performed immuno-precipitation assays using the gp41 TMD and mutant peptides, and the antimicrobial peptide LL37 as a negative control. LL37 was used due to its strong membrane affinity properties and lack of specific protein targets in macrophages. Figure 4D shows the strong precipitation of gp41 TMD with TLR2 compared to almost no precipitation of the gp41 mutant peptide. In order to validate that the inhibitory effect of the gp41 ENV peptide is due to inhibition of the interaction between the TMDs of TLR2 and TLR6, we performed a FRET competition assay between TLR2 and 6 in the absence (Figure S3 a in Text S1) or presence (Figure S3 b in Text S1) of the gp41 WT peptide. The data show that when the ENV peptide is present, the degree of association between the TLR peptides decreased from 40% FRET to 16%. This inhibition was further corroborated by immunoprecipitation of TLR6-TMD peptides with the TLR2 protein in the absence (Figure S1 c, left lane in Text S1) or the presence (Figure S1 c, right lane in Text S1) of the gp41 WT peptide.

### The Secondary Structures of the TMDs

In order to verify that the TMD peptides preserved helical structures, we used circular dichorism (CD) spectroscopy in a membrane mimicking solution (1% LPC). All three peptides exhibited α-helical secondary structures, typical of a TMD peptide (Figure S2 in Text S1). It is clear from the CD results that the gp41 mutant peptide (loss of the GxxxG motif) preserves a helical structure suggesting that any functional discrepancies are not as a result of major structural deformation.

### The ENV TMD Peptide Rescues Mice from LTA/GLN Mediated Sepsis

**Figure 5:**
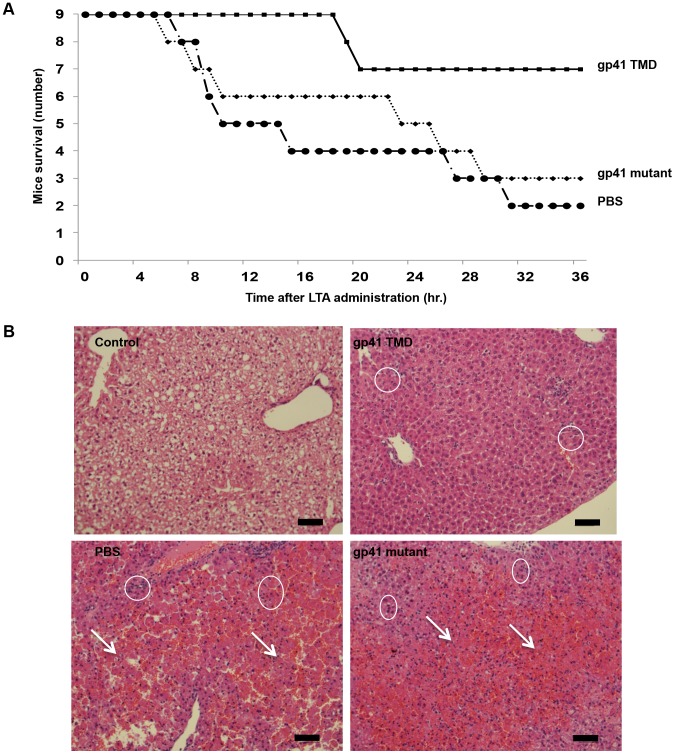
The gp41 TMD rescues mice in a LTA/GLN sepsis model and reduces liver damage. (**A**) Mice were inoculated with a lethal dosage of LTA/GLN and simultaneously treated with either the gp41 TMD peptide (▪) the gp41 mutant (♦) or PBS as control (•). Peptides were administrated at 5 mg mg/kg. (**B**) H&E staining of liver sections. Apoptotic foci are indicated in circles and arrows indicate hemorrhage. Cell death is multi focal as expected as a result of GLN treatment. Scale bar = 100 µm.

**Figure 6:**
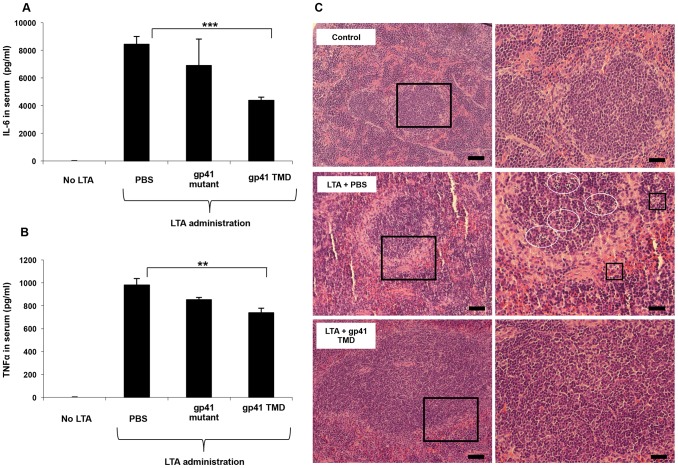
The gp41 TMD reduces serum cytokine levels in vivo and protects from consequential spleen damage. (**A–B**) Blood serum TNFα and IL-6 levels of PBS, gp41 TMD and gp41 mutant treated mice estimated 90 minutes and 210 minutes after LTA minutes and 210 minutes after LTA minutes after LTA/GLN injection (n = 9, ** - p<0.005, ***-p<0.0005). (**C**) H&E staining of spleen sections of control (top), LTA and no peptide (middle) and LTA and gp41 TMD treated (bottom) mice. Apoptotic foci in the white pulp are indicated in circles and red pulp as squares. Scale bars = 200 µm and 400 µm for left and right panels, respectively.

In order to corroborate the effect of the ENV TMD on TLR2 activation *in vivo* we utilized a murine model for acute sepsis caused by hyper-activation of TLR2 [13]. Mice were injected intraperitoneally (i.p) with 100 µg of LTA and 700 mg/kg D-galactosamine (GLN), and were either treated intravenous (i.v.) with gp41 TMD peptide (5 mg/kg), mutated peptide (5 mg/kg) or PBS. Upon injection of LTA/GLN, 7/9 PBS-treated mice and 6/9 of the gp41 mutant treated mice died while only 2/9 of the gp41 TMD treated mice died (Figure 5A). Histological analysis of the liver indicated that LTA/GLN treatment resulted in massive parenchyma damage associated with hemorrhage in both PBS and gp41 mutant treated mice (Figure 5B). However in the gp41 TMD treated samples, the tissue resembled that of control mice (Figure 5B) with no observable tissue damage or hemorrhage. This was also evident from the external feature of the tissues themselves, showing that mice not treated with the ENV TMD peptides had enlarged and inflamed livers and spleens (Figure S3 in Text S1). We analyzed serum levels of TNFα and IL-6 as these cytokines are secreted by resident macrophages and blood monocytes in the LTA/GLN model [13]. In accordance with the survival rates, our results indicate that the blood serum levels of TNFα and IL-6 were significantly lower for the gp41 TMD peptide-treated group compared to the untreated group (Figure 6A and B, respectively). We therefore examined spleen, in order to test activation of the immune system. The effect of an uncontrolled inflammatory response may lead to secretion of high levels of steroids, leading to massive cell death in the lymphocyte zones [25]. LTA/GLN injection resulted in a large number of apoptotic cell foci in the white pulp and congestion throughout the tissue in comparison to control mice spleens (Figure 6C middle and top panels, respectively). Comparably, gp41 TMD treatment decreased apoptosis to levels similar to the naïve samples (Figure 6C lower panel). Cell death was also detected in the red pulp of untreated mice and was absent from gp41 TMD treated mice. This result indicates that gp41 TMD decreased monocyte activation, resulting in less apoptosis in the spleen.

**Figure 7:**
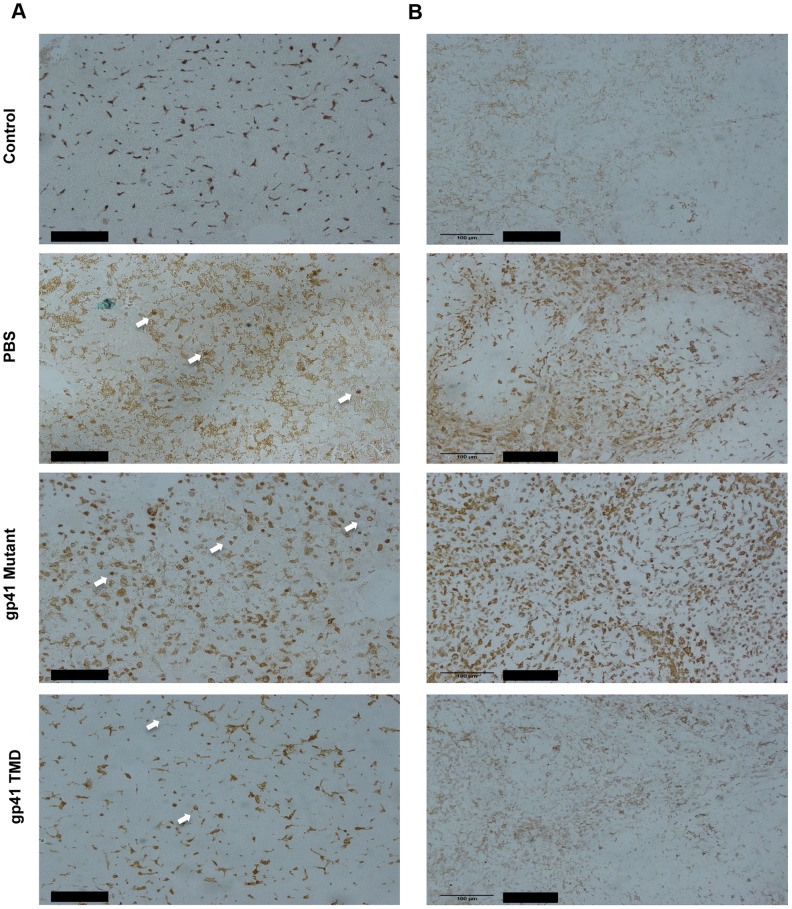
Gp41 TMD attenuates the activation of peripheral and tissue residential Macrophages in LTA/GLN treated mice. A) Representative data of 3 typical experiments of anti-Mac2 staining of the livers of LTA/GLN treated mice, treated with either PBS or WT gp41TMD or mutated gp41TMD. Arrows indicate round shaped macrophages that could be either infiltrating activated monocytes or activated kupffer cells. Scale bar is 100 µM. B) Representative Data of 3 typical experiments of anti-Mac2 staining of the spleens of LTA/GLN treated mice, treated with either PBS or WT Gp41TMD or mutated Gp41TMD. Scale bar is 100 µM.

In order to test the specific attenuating effect of gp41 TMD on LTA/GLN mediated sepsis, we tested whether the treatment resulted in an inhibitive effect on macrophages, as macrophages are the main immune cells that orchestrate this type of inflammation. We investigated whether the inhibitory effect of gp41 TMD peptide was tissue specific or systemic by staining both liver (tissue specific) and spleen (systemic) for the monocyte activation marker, Mac2 [26]. Liver staining showed that the administration of LTA/GLN resulted in increase in Mac2 positive cells, in addition to the appearance of round-shaped Mac2 positive cells that could be either activated residential macrophages (kupffer cells) that phagocysed dead hepatocytes, or infiltrated activated monocytes (Figure 7A, white arrows). The mutant gp41 peptide treated mice resembled the PBS+LTA/GLN treated mice and showed a similar increase in Mac2 positive cells and the appearance of the round shaped Mac2 positive cells, while WT gp41 TMD peptide treated mice resembled control non-LTA administrated mice, showing low intensity staining of Mac2 positive cells, and significantly decreased number of the round-shaped cells (Figure 7A). Similarly, spleen staining with anti-Mac2 of PBS+LTA/GLN treated mice and of a mutant gp41 TMD peptide +LTA/GLN showed very high elevation of Mac2 positive cells. However, mice treated with WT gp41 TMD+LTA/GLN showed minor amount of Mac2 positive cells that resembled the non-treated control mice (Figure 7B). These results suggest that the attenuating effect of Gp41 TMD was systemic rather than tissue specific, with high specific effects on monocytes and macrophages.

## Discussion

In this study, we report that the responsiveness of TLR2 is modulated by the ENV TMD. This inhibitory effect is driven through the direct association of the ENV TMD with the TLR2 TMD. Functionally, the ENV TMD inhibits TLR2 signaling in response to LTA and inhibits secretion of pro-inflammatory cytokines both *in vitro* and in an animal model of LTA/GLN sepsis.

Recent studies show that peptides derived from TMDs of several membrane proteins interfere with their assembly and function [23], [27], [28]. We utilized this strategy to assess the ability of ENV to affect TLR2 activation, and to identify the important amino acids within the TMD that contribute to this interaction. It has been shown that the TMD of TLR2 regulates its function in response to natural and synthetic ligands. The ENV TMD possesses a GxxxG motif, which is well defined as a membrane dimerization motif. Interestingly, the TLR2 TMD also possesses such a sequence (Figure S3, boxed in Text S1). The precise conformational changes of the TLR2 TMD that induce its dimerization with its partners leading to signal transduction are not known. Nevertheless, it is conceivable that the GxxxG motif of the TLR2 and TLR6 TMDs move in a piston mechanism (for an explanation on the piston motion, see Ref. [24]). This motion could explain how these motifs are important for the TLR function although they are not located parallel to each other within the membrane. Mutating the GxxxG motif significantly decreased the ability of the ENV TMD to inhibit macrophage responses to TLR2 ligands. This is expected because the peptides' mode of action is by inhibiting dimerization of TLR2 and TLR6 through interactions within the membrane milieu. Interestingly, the interaction of TMD sequences is not driven solely by the GxxxG motif, since the GpA TMD did not show any association with the TLR2 TMD. The specific nature of the interaction is demonstrated by the co-immunoprecipitation of TLR2 with the ENV TMD, while no interaction was detected with the mutated TMD. Overall, the data suggest that the ENV TMD interferes with the dimerization of TLR2/6 TMDs partially through their GxxxG motif. This subsequently leads to impaired complex signaling and to inhibition of cytokine secretion. The general effect of this inhibition is demonstrated by inhibition of the initial steps of signal transduction (ERK phosphorylation), as well as, by inhibition of an array of end point targets of TLR signaling (TNFα, IL-6 and MCP-1) each transcribed in a different complex of NFκB. Note that it has been shown that the GxxxG is important, but not sufficient, for helix-helix interactions within the membrane [21].

As CD4 and TLRs may reside in similar membrane environments [29], [30], and membrane-bound TLRs recognize viral glycoproteins, it is reasonable to assume that this leads to initiation of a response towards HIV-1 during fusion. Furthermore, activation of TLR signaling prior to HIV infection causes dendritic cells to be less permissive to infection [31]. Therefore, inhibiting the initial response towards the invading pathogen is a powerful tool to allow virus entry into the cell. An additional possible need for this inhibitory activity is at latent stages of viral infection. HIV-1 ENV and TLR2 both possess a membrane localization signal motif adjacent to their TMD (Figure S3 in Text S1). This signal motif determines the precise environment within the membrane in which the proteins reside. Although for T cells, ENV is expressed at low levels during latency, ENV expression levels on mononuclear phagocytes are high [32]. Several studies indicated the impairment of TLR signaling after viral infection and incorporation [5] which may assist in the establishment of infection by opportunistic bacteria and fungi, in addition to the loss of CD4^+^ T cells [33]. Together with our findings about the modulatory effect of ENV on TLR responses, this impairment may occur as a result of the interactions of the TMDs of ENV and TLRs, leading to the prevention of TLR activation upon ligand recognition. Noteworthy, several studies have linked the activation of TLR cascades to viral replication and burst. These studies focus on cells with established infection. This means that the virus successfully transmitted its genome into the cells and initiated the expression of its early genes [34]. In view of the small number of proteins expressed by the HIV genome, this immunosuppressive activity might exist with its fusogenic activity to help the virus to escape immune recognition and response towards it, prior to the activity of the viral early genes.

In summary, we demonstrate that the ENV TMD inhibits the activation of TLR2 signaling by interacting with its TMD. This property could be of great importance during fusion and serves as an additional mean of regulation of the activity of TLR at stages of Env expression. Our findings further demonstrate the complex strategies used by HIV-1 to manipulate the immune system. Interestingly, the ENV TMD is also involved in the late steps of the membrane fusion reaction. Such dual activity further demonstrates how viruses although expressing a limited number of proteins have evolved to alter so many cellular processes. Considering the strong ability of other gp41-derived segments to inhibit T-cell activation by various mechanisms, our study may indicate that these effects are also applicable to mononuclear phagocytes and additional targets should be further investigated. Apart from HIV pathogenesis, specific gp41 derived peptides may provide potential tools for treating uncontrolled immune responses, manifested here by sepsis.

## Materials and Methods

### Cell Culture

All *in vitro* assays were performed on RAW264.7 murine macrophages (ATCC- TIB71). Cells were grown at 37°C in the presence of 5% CO_2_ in DMEM supplemented with 10% FBS, L-glutamine, sodium pyruvate, non-essential amino acids, and antibiotics (Biological Industries, Beit-Haemek, Israel). For cytokine secretion measurements 2×10^5^ cells per well were cultured overnight in a 96-well plate. At the following day, media were replaced by fresh DMEM, including all supplements. Gp41 TMD peptides were dissolved in DMSO and added to the cells in different concentrations. The final concentration of DMSO was 1% for all groups. Cells were incubated with the peptide for 2 hours, washed and incubated with fresh media containing LTA. Cells were incubated with LTA for 5 hrs (TNFα) or 12 hours (IL-6, MCP-1) at 37°C, after which samples of the media were collected and stored at −20°C. Cytokine levels were evaluated using a mouse TNFα/IL-6 enzyme-linked immunosorbent assay kit (ELISAmax, Biolegend) according to the manufacturer's protocol. All experiments were done in triplicates.

### Gp41 and gp160 Intact Proteins Expression

Raw264.7 macrophages were transfected twice, 48 h and again 24 h prior to the experiment, either with p96ZM651gp160-CD5-opt plasmid for the expression of HIV envelope protein gp160, or with gp41-YFP expressing plasmid for the expression of gp41 HIV fusion protein [35]. To increase the expression rate we utilized TurboFect reagent (Thermo Scientific, Waltham, MA, USA) according to the manufacturer protocol. Surface expression of untagged gp160 was analyzed using immunofluorescence. Briefly, cells were washed twice with PBS and fixed with 4% paraformaldehyde for 15 min at room temperature. Antibody staining was conducted using a goat polyclonal anti-HIV-1 gp120-biotin conjugated antibody and a ATTO 488 conjugated, goat IgG (H&L) antibody. Fluorescence intensities were obtained using an inverted FluoView 1000 confocal microscope (Olympus, Tokyo, Japan) with a 60× oil immersion objective (numerical aperture 1.35) at 25°C with a frame size of 512×512 pixels. ATTO 488 was excited with a laser diode at 488 nm and detected between 500 and 600 nm.

### Fluorescence Resonance Energy Transfer (FRET) between NBD and Rhodamine (TAMRA)-Labeled Peptides

Large Unilamellar Vesicles (LUVs) composed of phosphatidylcholine (PC) and cholesterol (Chol) (9∶1, w/w) were prepared using the extrusion method as described previously [36]. The fluorescence experiments were performed with pairs of peptides using 4-Fluoro-7-nitrobenzofurazan (NBD, BioChemika) -labeled peptides as donors and rhodamine-labeled peptides as acceptors and as described previously [13]. Maximum FRET values were obtained by calculating the changes in the emission of the donor peptide at 530 nm.

### Secondary Structure Determination Utilizing Circular Dichroism (CD) Spectroscopy

CD measurements were performed by using an Applied Photophysics spectropolarimeter. The spectra were scanned using a thermostatic quartz cuvette with a path length of 1 mm. Wavelength scans were performed at 25°C; the average recording time was 15 s, in 1 nm steps, through the wavelength range of 195–260 nm. Peptides were scanned at a concentration of 50 µM in buffer only (HEPES 5 mM) or in a membrane mimetic environment of 1% Lysophophatidilcholine (LPC) in buffer.

### Construction of the GALLEX Chimera

The hetero-interactions of the TMDs within a natural membrane environment were studied using the GALLEX assay [37]. The expression vectors and E. coli strains required for the assay were kindly provided by Dr. Dirk Schneider. Briefly, Two plasmids are used in the assay containing either the wild-type LexA sequence (pBLM, encoding for resistance to Tetracycline) or a mutated form of LexA (pALM, encoding for resistance to Ampicillin). The desired TM sequences are inserted between these two protein sequences using standard cloning techniques. The resulting plasmids were both transformed into an SU202 E. coli strain, which then expressed the LexA-TM-MalE fusion proteins (after induction with 1 mM IPTG). Hetero-association of TM domains leads to the formation of LexA_WT_/LexA_mut_ heterodimers that bind to a hybrid LexA promoter/operator in the reporter strain SU202 (that recognizes the mutant LexA domain), and repress the expression of β-gal. Furthermore, as the hybrid promoter is designed to bind only the wt and mutant heterodimers, it will not recognize wild-type LexA homodimers. Thus, TM homo-oligomer formation does not interfere with the measurement of hetero-association. The TMDs of GlycophorinA (GpA) and its G83I mutant, used as positive and negative controls, respectively, are ITLIIFGVMAGVIGTILLI and ITLIIFGVMAIVIGTILLI. All the constructs were confirmed by DNA sequencing.

### Measurements of Dimerization (β-Galactosidase Assay)

The dimerization propensity of the two constructs was measured by transforming an E. coli SU202 indicator strain with the relevant pALM and pBLM plasmids (each harboring a different TM sequence) simultaneously. The bacteria were grown overnight in the presence of 1 mM IPTG in LB medium with ampicillin and tetracycline in a 24-well plate. 10 µL of bacteria were resuspended in 100 µL of Z-buffer (60 mM Na_2_HPO_4_, 40 mM N_a_H_2_PO_4_, 10 mM KCl, 1 mM MgSO_4_, pH = 7.0, 10% chloroform, 1% β-mercaptoethanol), and the absorbance (OD_600_) was measured. Bacteria were lysed with 50 µL of 2% SDS in Z-buffer. O-nitrophenyl-galactopyranose (ONPG) was added and a kinetic reading of absorbance at OD_405_ was performed for 20 min to measure the activity of β-gal. Vmax was calculated and divided by the absorbance at OD_600_ to correct for bacterial growth. Strong dimerization results in strong inhibition of β-gal expression and less color formation.

### Western Blot Analysis

Cell lysates were prepared in radioimmunoprecipitation (RIPA) assay buffer (50 mM Tris HCl pH 7.5, 150 mM NaCl, 1% Nonidet P-40, 0.5% sodium deoxycholate, 0.1% SDS) containing 50 mM NaF, 2 mM Na3VO4, protease and phosphatase inhibitors (Sigma-Aldrich). Protein concentration was measured using the BCA Protein Assay Kit (Pierce Chemical Co.). Fifty µg of protein was loaded and separated on 8–15% SDS-PAGE and transferred to a nitrocellulose membrane. The membrane was blocked using 5% bovine serum albumin (BSA) in phosphate-buffered saline containing 0.1% Tween-20 (PBST) for 1 h at room temperature. The primary antibody was diluted in PBST containing 1% BSA and incubated with the membrane at 4°C overnight. After 3 washes with PBST, membranes were incubated with the secondary antibody in PBST containing 1% BSA at room temperature for 1 h.

### qPCR Gene Expression

The following sequences were used for quantitative mRNA analysis:

TNFα Forward: CTTGTGGCAGGGGCCACCAC

  Reverse: CCATGCCGTTGGCCAGGAGG

MCP-1 Forward: TCACCTGCTGCTACTCATTCACCA

  Reverse: AGCACAGACCTCTCTCTTGAGCTT

### Antibodies and Reagents

Anti phospho-ERK1, 2 and anti-total ERK1, 2 were purchased from Sigma-Aldrich and anti GAPDH was purchased from Millipore. Goat polyclonal anti-HIV-1 gp120-biotin conjugated antibody was purchased from Abcam (Cambridge, United Kingdom). ATTO 488 Conjugated Goat IgG (H&L) antibody was purchased from Rockland (Gilbertsville, PA, USA). *S. aureus* LTA was purchased from Sigma-Aldrich and D-galactosamine was purchased from Calbiochem. TNFα and IL-6 ELISA kits were purchased from Biolegend. The plasmid p96ZM651gp160-CD5-opt was obtained through the NIH AIDS Reagent Program, Division of AIDS, NIAID, NIH: from Drs. Yingying Li, Feng Gao, and Beatrice H. Hahn. The plasmid gp41-YFP, provided by Roland Schwarzer encodes a HIV-1 gp41 fusion protein with the c-terminal external parts of the protein replaced by a Yellow Fluorescent Protein.

### LTA Induced Sepsis-Like Model

Animal studies were carried out in strict accordance with the Israeli law and the National Research Council guidelines. All animal experiments were conducted at the Weizmann Institute of Science and approved by the Weizmann Institutional Animal Care and Use Committee (IACUC permit no. 01190107-4). 12- week-old C57 Black female mice were injected IP with 50 mg/kg of *S.aureus* LTA and 800 µg/kgof D-galactosamine in PBS, as previously described [13], in combination with 5 mg/kg of gp41-TMD or mutated gp41-TMD or PBS, and monitored for survival for 36 hours. Blood samples were collected for TNFα and IL-6 serum levels measurements. Serum was extracted by blood coagulation in RT followed by spin down centrifugation. Serum samples were analyzed by ELISA assays.

### Histological Analysis

Spleen and liver samples were collected either post mortem or from sacrificed mice 36 hours after the injection of LTA/GLN and kept in formaldehyde (4%) for 18 hours and embedded in paraffin blocks. 4 µm sections were then prepared and stained with hematoxylin and eosin by standard protocols.

### Peptide Synthesis and Purification

Peptides were synthesized by a 9-fluorenylmethoxylcarbonyl (Fmoc) solid-phase method on Rink amide MBHA resin (Calbiochem-novabiochem, San Diego, California) by using an ABI 433A automatic peptide synthesizer (Applied Biosystems, Foster City, CA). Peptide synthesis was followed by cleavage from the resin by incubation for 3 h with 95% TFA, 2.5% H_2_O, and 2.5% triethylsilane, followed by purification by RP-HPLC (>98%) on a Vydac C4 column (Grace Discovery Sciences, Deerfield, Il), and then identification by electro-spray mass spectroscopy. The following fluorophores were used for fluorescent labeling: 4-Fluoro-7-nitrobenzofurazan (NBD, BioChemika) and 5(6)-Carboxytetramethylrhodamine *N*-succinimidyl ester (TAMRA, BioChemika). Peptides used for in vivo experiments were treated twice with 20% acetic acid in order to replace the trifluoroacetate anion added during HPLC purification.

## Supporting Information

Text S1 This file contains Table S1 and Figures S1–S5. **Figure S1**. This figure shows that gp41 TMD disrupts the interaction between TLR2 and TLR6 as revealed by Fluorescence Resonance Energy Transfer (FRET) measurements. In this experiment the NBD-labeled TLR2 TMD peptide was added first from a stock solution in DMSO (final concentration 0.1 µM and a maximum of 0.25% (v/v) DMSO) to a dispersion of PC∶Chol LUVs (100 µM) in PBS. This was followed by the addition of the following: **A**. Rhodamine labeled TLR 6 TMD or in addition with un labeled WT gp41 (**B**), in sequential doses ranging from 0.01 µM to 0.1 µM (stock in DMSO), generating a ratio of 1∶10, 1∶5, 1∶2 and 1∶1 Rhodamine∶NBD presented from top to bottom. In both graphs, the upper spectrum represents the emission of the NBD-labeled peptide alone. (**C**) IP of TLR2 from raw264.7 cells in the presence of the indicated Rho-labeled peptides. The result of the last lane from the marker shows that in the presence of WT gp41 there is a decrease in the binding of the TLR6-TMD peptide to the TLR2 protein. **Figure S2.** This figure shows the secondary structure of the peptides used in this study as measured by CD spectroscopy. Spectra were measured at 10 µM in a 1% LPC solution. Graphs are the mean of 3 measurements. **Figure S3.** This figure shows that treatment with gp41 WT peptide rescues mice organs form inflammatory related damage. In this experiment tissue samples of liver (A) and spleen (B) from mice taken at the experimental end point. In both tissues inflammation is evident by the tissue color. **Figure S4.** This figure shows **s**equence alignment of the TMDs of gp41 (top) and TLR2 (bottom). Arrows indicate the predicted TMD. Underline indicates the localization motif to cholesterol enriched regions within the membrane. **Figure S5.** This figure demonstrates that gp160 and gp41 infected RAW cells show a membranous expression of gp160 and gp41. (A) Representative results of cells infected with gp160 plasmid (see materials and methods) that were stained with anti-gp160 antibody without permeabilization. Green indicates membranous gp160. Scale bar is 20 µm. (**B**) Representative data of RAW cells expressing GFP-gp41 chimera protein (see materials and methods). Scale bar is 40 µm. (**C**) A representative image of confocal microscopy analysis of GFP-gp41 expressing RAW cells showing membranous expression of the chimeric protein. Scale bar is 10 µm. (**D**) Representative data of FACS analysis of GFP-gp41 RAW transfected cells.
(DOCX)
