# Correction: The HIV-1 Envelope Transmembrane Domain Binds TLR2 through a Distinct Dimerization Motif and Inhibits TLR2-Mediated Responses

**DOI:** 10.1371/journal.ppat.1004408

**Published:** 2014-09-02

**Authors:** The PLOS Pathogens Staff

The fourth author's name is spelled incorrectly. The correct name is: Roland Schwarzer. The correct citation is: Reuven EM, Ali M, Rotem E, Schwarzer R, Gramatica A, et al. (2014) The HIV-1 Envelope Transmembrane Domain Binds TLR2 through a Distinct Dimerization Motif and Inhibits TLR2-Mediated Responses. PLoS Pathog 10(8): e1004248. doi:10.1371/journal.ppat.1004248
