# Supplementary material for: The HIV-1 Envelope Transmembrane Domain Binds TLR2 through a Distinct Dimerization Motif and Inhibits TLR2-Mediated Responses
Source: PLoS Pathog. 2014 Aug 14;10(8):e1004248. doi: 10.1371/journal.ppat.1004248 (PMC4133399; doi:10.1371/journal.ppat.1004248)
Supplement: Text S1 — This file contains Table S1 and Figures S1–S5. Figure S1. This figure shows that gp41 TMD disrupts the interaction between TLR2 and TLR6 as revealed by Fluorescence Resonance Energy Transfer (FRET) measurements. In this experiment the NBD-labeled TLR2 TMD peptide was added first from a stock solution in DMSO (final concentration 0.1 µM and a maximum of 0.25% (v/v) DMSO) to a dispersion of PC∶Chol LUVs (100 µM) in PBS. This was followed by the addition of the following: A. Rhodamine labeled TLR 6 TMD or in addition with un labeled WT gp41 (B), in sequential doses ranging from 0.01 µM to 0.1 µM (stock in DMSO), generating a ratio of 1∶10, 1∶5, 1∶2 and 1∶1 Rhodamine∶NBD presented from top to bottom. In both graphs, the upper spectrum represents the emission of the NBD-labeled peptide alone. (C) IP of TLR2 from raw264.7 cells in the presence of the indicated Rho-labeled peptides. The result of the last lane from the marker shows that in the presence of WT gp41 there is a decrease in the binding of the TLR6-TMD peptide to the TLR2 protein. Figure S2. This figure shows the secondary structure of the peptides used in this study as measured by CD spectroscopy. Spectra were measured at 10 µM in a 1% LPC solution. Graphs are the mean of 3 measurements. Figure S3. This figure shows that treatment with gp41 WT peptide rescues mice organs form inflammatory related damage. In this experiment tissue samples of liver (A) and spleen (B) from mice taken at the experimental end point. In both tissues inflammation is evident by the tissue color. Figure S4. This figure shows sequence alignment of the TMDs of gp41 (top) and TLR2 (bottom). Arrows indicate the predicted TMD. Underline indicates the localization motif to cholesterol enriched regions within the membrane. Figure S5. This figure demonstrates that gp160 and gp41 infected RAW cells show a membranous expression of gp160 and gp41. (A) Representative results of cells infected with gp160 plasmid (see materials and methods) that were [file ppat.1004248.s001.docx]

**Supplementary Material**

**Text S1**

**The HIV-1 Envelope Transmembrane Domain Binds TLR2 Through a Distinct Dimerization Motif and Inhibits TLR2-Mediated Responses**

Eliran Moshe Reuven, Mohammad Ali, Etai Rotem, Roland Schwarzter, Andrea Gramatica, Anthony H. Futerman and Yechiel Shai

**Figure S1**

**Figure S2**

**Figure S3**

F**igure S4**

**Figure S5**
